# A Network Pharmacology Study on the Active Ingredients and Potential Targets of* Tripterygium wilfordii* Hook for Treatment of Rheumatoid Arthritis

**DOI:** 10.1155/2019/5276865

**Published:** 2019-04-15

**Authors:** Wei Hu, Wanjin Fu, Xin Wei, Yang Yang, Chao Lu, Zeyuan Liu

**Affiliations:** ^1^Department of Clinical Pharmacology, Affiliated Hospital of Academy of Military Medical Sciences, Beijing, China; ^2^Department of Pharmacology, The Second Hospital of Anhui Medical University, Hefei, Anhui, China

## Abstract

Traditional Chinese medicine has specific effect on some chronic diseases in clinic, especially in rheumatic diseases.* Tripterygium wilfordii* Hook (TWH) is a traditional Chinese medicine commonly used in the treatment of rheumatoid arthritis (RA); the unique therapeutic effect has been confirmed by a large number of research papers. TWH has many compounds that lead to its active compounds. However, the potential targets and pharmacological and molecular mechanism of its action treatment of rheumatic diseases are not entirely clear. Therefore, the network pharmacology approach is needed to further study and explore its treatment mechanism. We have successfully set up 10 networks, including four major networks and other networks. Four major networks include rheumatoid arthritis disease network, compound-compound target network of TWH, TWH compound target-rheumatoid arthritis disease network, and TWH-rheumatoid arthritis disease-mechanism network. Other networks consist of RA disease and TWH related targets clusters, biological processes, and pathways network. Our study successfully predicted, explained, and confirmed the TWH of RA disease molecular synergy and found the potential of RA related targets, cluster, biological process, and pathways. This study not only provides prompts to the researcher who explores pharmacological and biological molecular mechanism of TWH applying to RA disease, but also proves a feasible method for discovering potential activated compounds from Chinese herbs.

## 1. Introduction 

Rheumatoid arthritis (RA) is a long-term autoimmune disorder with a global incidence of 1 percent and most common in middle-aged women, characterized by warm, pain, swollen, and stiffness of peripheral joints; it may result in progressive joint damage, deformity, dysfunction, and increased mortality [1]. In severe cases, the joints are fibrous or bony, and they lose their joint function due to the atrophy and spasm of the surrounding muscles and lead to patients not being able to live by themselves. In addition to joint symptoms, external or visceral lesions can occur, such as rheumatoid nodules, heart, lung, kidney, peripheral nerves, and eye [2].

Currently, the commonly used treatment ways in RA are surgery, medicine, and psychotherapy. Drugs used to treat RA are classified into nonsteroidal compounds (NSAIDS), disease-modifying antirheumatic drugs (DMAKDS), and glucocorticoids [3].

These medications may prevent or reduce joint damage and help to maintain regular structure and function of the joint. However, these drugs can only control the disease progress of RA and usually associated with many adverse effects such as methotrexate pteridine, DMARDs drugs; its side effects include nausea, vomiting, and digestive tract reaction, oral inflammation, skin rashes, hair loss, bone marrow suppression, and liver toxicity; occasionally occurring can lead to death of pulmonary interstitial lesions, and glucocorticoids drugs are associated with osteoporosis and infection due to their effects on hormones [4]. NSAIDS drugs can cause gastrointestinal reactions, which can cause gastrointestinal perforation and bleeding, bone marrow suppression, and other side effects [5]. With the development of medicine and pharmacy technology, RA therapy has entered a multistage comprehensive treatment stage. Complementary and alternative medicine (CAM) becomes the patient's choice. Traditional Chinese medicine (TCM) as an important part of CAM, has been the most frequently used alternative treatments for RA [6]. In the world, including developed countries, TCM has been widely used in the prevention and control of various chronic diseases in recent years [7].

*Tripterygium wilfordii *Hook (TWH) was recorded in the compendium of materia medica, written by Li Shizhen, one of the most important traditional Chinese medicine classics and is a famous medicine for the treatment of RA. According to the theory of Chinese medicine, this medicine can resist rheumatism, activate blood circulation, and reduce swelling and pain. It is commonly used in the treatment of RA and ankylosing spondylitis and other diseases. Its antirheumatic effect is inferior to other steroid drugs and is superior to other antirheumatic Chinese and western medicines. It can replace most of the steroids for treatment, reducing its dependence and dosage with no rebound phenomenon after drug withdrawal [8, 9]. However, its pharmacological mechanism has not yet been fully elucidated.

**Figure 1:**
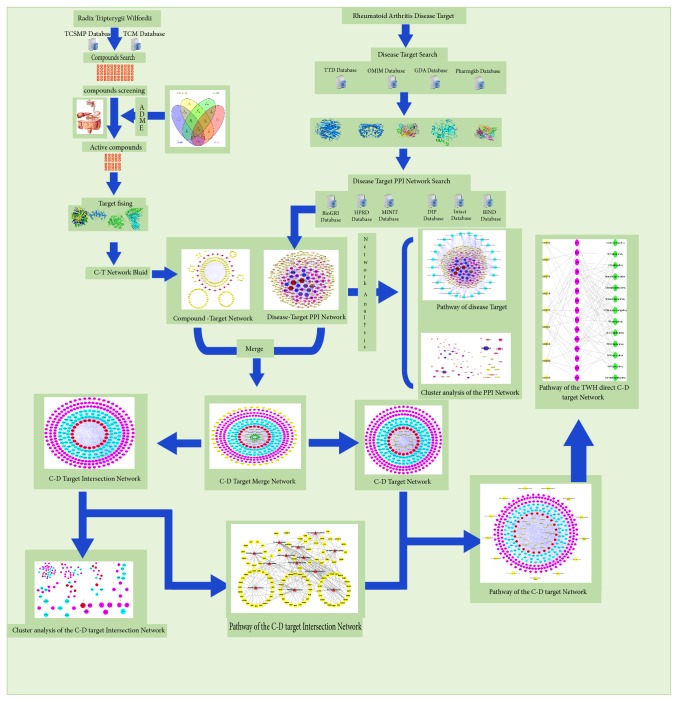
TWH treatment of RA network pharmacological flow chart.

Due to the multichemical components, multipharmacological effects and multiaction targets of TCM in the treatment of diseases, the traditional research methods are difficult to entirely explain the mechanism of action [10]. However, the network pharmacology produced by the integration of bioinformatics and pharmacology in recent years can clearly make clear the principle of action of such drugs. Network pharmacology can systematically explain the role of multicomponent drugs in the treatment of diseases by finding the relationship between the drug components with targets and targets with diseases, and abstracting them into network relationships models [11, 12]. Therefore, we will use the method of network pharmacology to study the effect of TWH on the treatment of RA and expect to find its medicinal value. The workflow of the study on TWH against RA based on network pharmacology was shown in Figure 1.

## 2. Materials and Methods

### 2.1. Data Preparation

#### 2.1.1. Composite Compounds of Tripterygium wilfordii Hook (TWH)

All the composite compounds of TWH were obtained from the following database: TCM database@Taiwan (http://tcm.cmu.edu.tw/), The Traditional Chinese Medicine Systems Pharmacology Database (TcmSPT, http://lsp.nwu.edu.cn/index.php), and literature review. In total, the information of 144 TWH compounds was collected [13]. The detailed information on the composite compounds of TWH is described in Table S1.

#### 2.1.2. ADME Screening of TWH

Compared to traditional research methods to screen effective compounds, it is faster and more economical to screen active chemicals using ADME (absorption, distribution, metabolism, and excretion) models simulated in silico systems [14]. We used an in silico integrative model ADME to identify the potential bioactive compounds of TWH, including the evaluation of Caco-2 permeability, oral bioavailability (OB), and drug-likeness (DL), and (half-life) HL were used to apply ADME-related models.

Oral bioavailability (OB): the OB value is calculated on silicon system using the OBioavail 1.1 model to calculate the relationship between the compound and the P4503A4 enzyme and the transporter (p-glycoprotein). The OB value reflects the ability of compounds to enter the human circulatory system [15]. We selected compounds with an OB value greater than 30% as the screening target, and obtained a total of 75 compounds. The results are described in Table S2.

Caco-2 permeability: absorption of the drug in the intestine is closely related to its permeability in the intestinal epithelial cells. The human intestinal cell line Caco-2 is often used as an effective in vitro model to study the passive diffusion of drugs through the intestinal epithelium [16]. We screened for Caco-2>- 0.4 of 108 potentially well-absorbed compounds obtained. The results are described in Table S3.

Drug-Like value: the DL value is used for the ability of a prospective compound to become a drug by calculating the similarity between a potential compound and a known drug. Drug-like compounds are not drugs, but they have the potential to become drugs. Such compounds are called drug-like molecules or drug analog molecules [17]. We used traditional Chinese medicines of TCM DL>0.18 as selection criteria and obtained a total of 107 potential compounds. The results are described in Table S4.

Drug half-life refers to the concentration of the drug in the blood or the amount of drug in the body reduced to one-half, for the important properties of the drug; you can calculate the dosing interval, determine the dose, and calculate the volume of drug accumulation [18]. The simulation calculation method of drug half-life is described in detail in a novel Systems Pharmacology model for herbal medicine injection: a case using Reduning Injection [19]. We chose a compound with a half-life of 3 hours and obtained 58 potential compounds. The results are described in Table S5.

**Figure 2:**
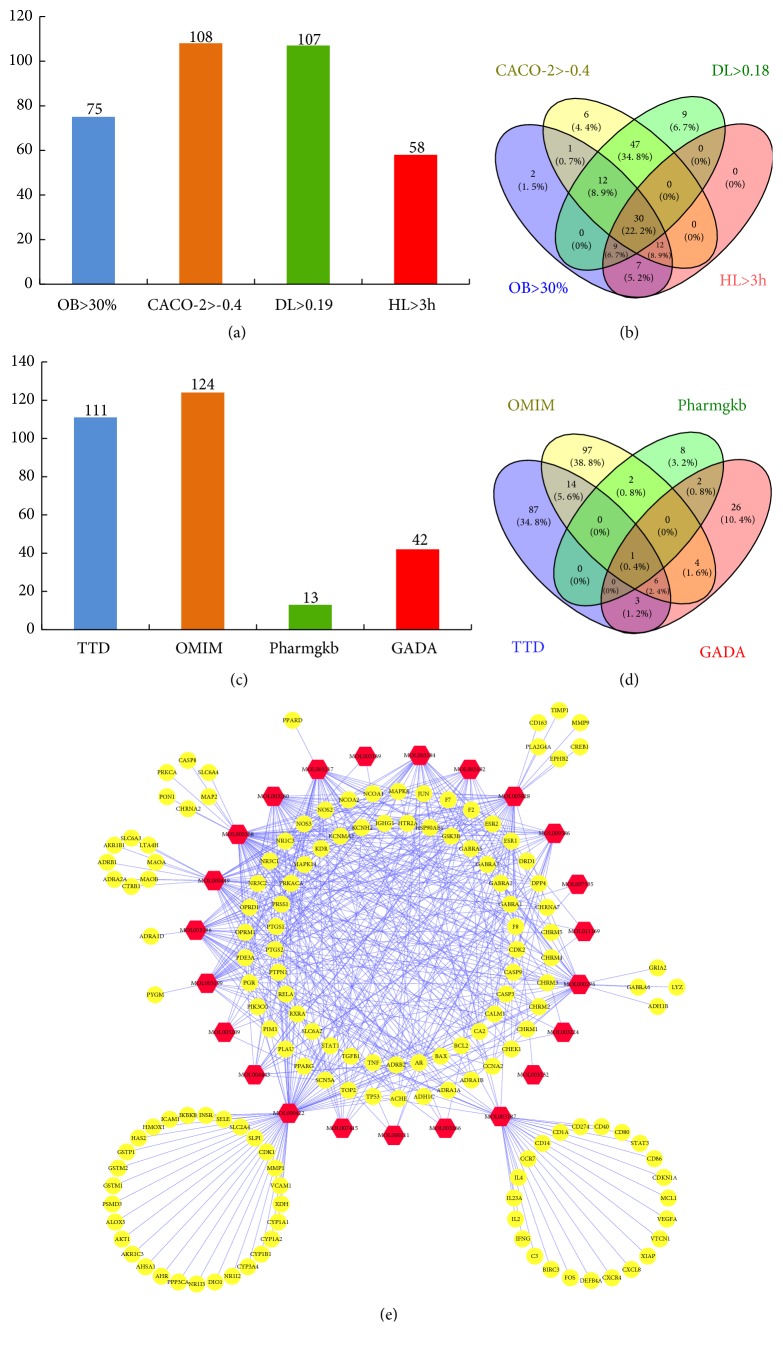
TWH active compound AMDE screening and TWH compound-compound target network. (a) The active compound according to the OB, CACO-2, DL, HT four index values screening results. (b) The Venn diagram of the active compound according to the four index values of OB, CACO-2, DL, and HT. (c) The retrieval results of RA targets for different disease databases. (d) The Venn diagram of the retrieval results of RA targets for different disease databases. (e) TWH compound-compound target network of YHD consists of 150 compound targets and 23 compounds (red hexagonal represents compound; yellow circle represents target).

By screening TWH-combined OB, Caco-2, DL, and HL indicators, 30 potentially drug-producing compounds were finally obtained (Figures 2(a) and 2(b)). The detailed information on the composite compounds of TWH is described in Table S6.

#### 2.1.3. Prediction of Targets of TWH

All the active ingredients were input into Traditional Chinese Medicine Systems Pharmacology Database (http://lsp.nwu.edu.cn/tcmsp.php), which is a unique systematic pharmacology platform of Chinese herbal medicines that captures the relationships between drugs and targets [20]. Since the obtained target includes various species of biological targets and the relationship between the more concise expressed ingredients and the target, all targets names are put into uniport sites (http://www.uniprot.org/) to search for target genes names being selected by human species.

#### 2.1.4. Search Targets of RA Diseases

We collected the therapeutic target data for the treatment of arthritis from four resources: (1) Therapeutic Target Database (TDD) (https://db.idrblab.org/ttd/); (2) Online Mendelian Inheritance in Man(OMIM) (http://omim.org/); (3)PHARMGKB (https://www.pharmgkb.org/); and (4) Genetic Association Database (GADA)(https://geneticassociationdb.nih.gov/) [21]. In these databases, we used the keywords “rheumatoid arthritis” as the queries to search known therapeutic targets of diseases. Through the above search, we obtained 13 genes in the PHARMGKB database, 42 genes in the GADA database, 111 genes in the TTD database, and 124 genes in the OMIN database in which all genes are associated with RA. These genes from databases were, respectively, put into the Venny 2.1 system (http://bioinfogp.cnb.csic.es/tools/venny/index.html) which is a professional tool for producing the Venn diagram and acquired 250 different genes related to RA (Figures 2(c) and 2(d)). The results are described in Table S7.

#### 2.1.5. Protein–Protein Interaction (PPI) Data

We used the Bisogenet3.0 plug-in in Cytoscape3.6 to look for the Protein–protein interaction (PPI) data. Bisogenet is a very powerful bioinformatics plug-in that directly accesses 6 open biological databases on protein-protein interactions [22]. When we use Bisogenet to get the protein interaction relationship, its main parameters are set as follows. (1) Set the “Organism” option to “Homo sapiens” in the Identifiers setting. (2) Set the “Biorelation types in the Data” setting to DIP, BIOGRID, HPRD, INTACT, MINT, BIND. (3) Select in the Method setting. A criteria to build the network is set to “Input nodes only”. (4) Output settings set Represent network nodes in term of “Genes”. After getting the PPI data, we have removed all the self-interacting protein data in order to better display PPI. The information of disease database is described in Table S8.

### 2.2. Network Construction

#### 2.2.1. Network Construction Method

The following networks were constructed: (1) rheumatoid arthritis disease network; (2) compound-compound target network of TWH; (3) TWH-rheumatoid arthritis network; (4) TWH- rheumatoid arthritis disease-mechanism network.

All the networks can be generated via utilizing the network visualization software Cytoscape [23] (version: 3.6.0, http://cytoscape.org/). Cytoscape is a free and open-source software platform commonly used to visualize molecular interaction networks and biological pathways and also can integrate these networks with annotations, gene expression profiles, and other state data. In addition, it has a variety of tools for comprehensive analysis of bioinformatics networks to obtain the key nodes, which conveniently help us to get the main mechanisms of traditional Chinese medicine to treat diseases.

#### 2.2.2. Cluster

Some proteins in biological activities have a close relationship and have the same or similar functions, which can be defined as a cluster. They play an important role in the development of the disease. Owing to the topology module, function module and disease module have the uniform meaning in the network, the functional module is the same as the topology module, and the disease can be regarded as interference and destruction of the functional model. MECODE is a plugin in Cytoscape software that can help us to get the protein clusters from the complex bioinformatics network [24].

### 2.3. Enrichment Analysis

We applied the Database for Annotation, Visualization and Integrated Discovery (DAVID) to carry out GO enrichment analysis and pathway enrichment analysis of proteins in PPI network [25].* P* values were derived from the DAVID database and are modified Fisher exact* P* values. Smaller* P* values indicated greater enrichment. Only functional annotations having the enrichment* P* values corrected by both algorithms Bonferroni and Benjamini (P < 0.05) were chosen for further analysis.

## 3. Results and Discussion

### 3.1. Compound-Compound Target Network Analysis

This network is composed of 173 nodes (150 compound target nodes and 23 compound nodes) and 518 edges. In the compounds-targets network we can see that some compounds can act on multiple physiological targets (e.g., kaempferol compounds act on 72 potential targets, beta-sitosterol compounds act on 55 potential targets, Stigmasterol compounds act on 43 targets, etc.). Some compounds only act on a few targets; for example, Triptofordin B1 compound only regulates ESR1. At the same time, some targets can be regulated by multiple compounds, which may play important roles in the treatment of diseases. For instance, ESR1, AR, F2, and PTGS2 are regulated by over 60% of potentially active compounds. Some targets are only regulated by one compound, such as ADH1B, ADRA1D, and AHR targets. The network details are provided in Figure 2(e) and Tables S9 and S10.

### 3.2. Rheumatoid Arthritis Network Analysis

#### 3.2.1. Rheumatoid Arthritis Network

The nodes in the disease PPI network represent the interrelationships during the development of the RA. We constructed a total of 250 nodes and 633 edges in the PPI network of RA. The area size of the node is positively related to the degree of the node, and degree indicates the number of relationships between the node and other nodes in the network. The areas of nodes such as PIK3R1, IKBKG, FN1, LCK, and LYN are larger and can be easily found in the PPI network; the corresponding degree values of each node are 37, 30, 29, 28, 27, and 26. These targets may play a key role in the development of RA disease. The network details are provided in Figure 1S and Tables S11 and S12.

#### 3.2.2. Clusters of Rheumatoid Arthritis Network

By using the MCODE plug-in cluster analysis of the PPI network of RA disease, seven clusters were obtained (for cluster analysis of specific parameters, please refer to Table 1 and Figure S2), and enrichment analysis was performed with clusters scores > 2 targets and screened with a Term* P* value < 0.001. Analyzing another cluster by the same way, we get the same related biological processes, molecular functions, and cell components. The details are described in Table S13.

#### 3.2.3. Pathway of Rheumatoid Arthritis Network

**Figure 3:**
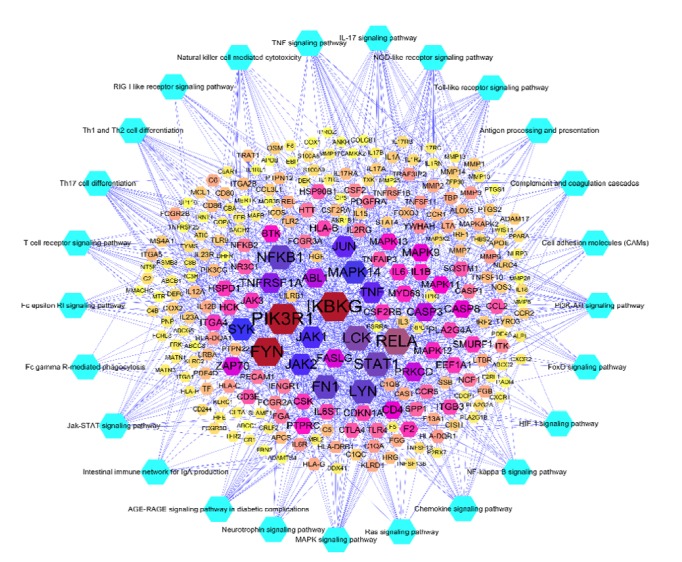
Pathway of rheumatoid arthritis disease target.

A total of 25 pathways related to RA were obtained by placing RA-related genes in DAVAID for pathway analysis. PI3K-Akt signaling pathway contains 33 genes, which is the most; IL-17 signaling pathway has 32 genes; Jak-STAT signaling pathway, NOD-like receptor signaling pathway and TNF signaling pathway are 26 genes in the same number; Toll-like receptor signaling pathway includes 25 gens; MAPK signaling pathway is 22 and so on. We obtained the disease target pathway network by combining the pathway-related gene information with the RA disease PPI network. This network concisely expresses the interaction of disease targets and the relationship between pathways and targets (Figure 3). This network includes 228 nodes (25 pathway nodes and 250 gene nodes), and 111 edges. RA disease pathway details are provided in Tables S14 and S15.

These pathways associated with RA-related genes may become the key pathway for the development of RA. At the same time, we found that some important genes act on many pathways (e.g., IKBKG, TNF, PIK3R1, and MAPK11 genes act on more than 5 pathways). These pathways can regulate complex biological and metabolic processes. Interfering with certain targets of these pathways may be a potential strategy for the future treatment of RA diseases.

RA is an autoimmune disease characterized by chronic inflammation of the synovial membrane. Its main pathways include PI3K-Akt signaling pathway, IL-17 signaling pathway, TNF signaling pathway, MAPK signaling pathway, and T cell receptor signaling pathway, NF-kappaB signaling pathway, Fc epsilon RI signaling pathway, Neurotrophin signaling pathway, Ras signaling pathway, RIG-I-like receptor signaling pathway, FoxO signaling pathway, and HIF-1 signaling pathway. These pathways are closely related to the balance between anti-inflammatory factors and inflammatory factors, inflammatory cell migration, infiltration, cartilage extracellular matrix degradation and cell proliferation, differentiation, and apoptosis. Clinically used immunological preparations such as TNF-*α* inhibitor, B-cell depletion and blocking antibodies, IL-6 inhibition, and IL-1 inhibition can affect RA diseases by influencing these pathways.

### 3.3. TWH-Rheumatoid Arthritis Network Analysis

#### 3.3.1. TWH's C-D Target Merge Network

**Figure 4:**
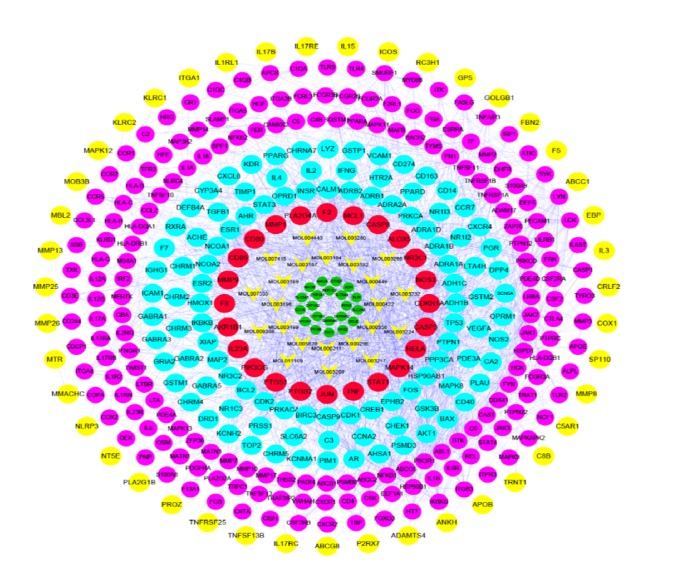
TWH-RA disease network (green circle represents having nothing to do with the RA disease targets, star on behalf of TWH compounds, red circle represents compounds directly affect the RA targets, blue circle represents compound indirect role in RA targets of targets, purple is on behalf of TWH indirect targets, and yellow circle represents having nothing to do with the TWH targets).

TWH's C-D Target Merge Network is obtained by merging the disease PPI network with a drug target network. The C-D Target Merge Network directly and concisely expresses the relationship between multitargets of multicompounds, as well as presenting the relationship between multicompound targets and disease targets in one figure (Figure 4). The network consists of a total of 399 nodes and 1812 edges. The nodes on the network diagram are divided into 6 categories from the inside out, and they are expressed in different shapes or colors. The classification from the inside to outside is as follows. A total of 12 nodes of the first type are drug targets that are not related to disease targets. A total of 23 second-type nodes are drug compounds. A total of 24 nodes of the third type are the target of the direct action of drug compounds. A total of 162 types of the fourth type of nodes are indirect effects of drug targets and disease targets. A total of 152 nodes of the fifth category are disease targets that have indirect effects on drug targets. A total of 43 nodes of the sixth category are disease targets that have no effect on drug targets. The network details are provided in Figure S3 and Tables S16 and S17.

In order to better analyze the mechanism of TWH's action on RA disease, we have deleted the drug compounds and indirect targets in the TWH-RA network to obtain the TWH-RA's C-D target intersection network. The C-D target intersection network consists of 118 nodes and 667 edges. We performed clustering and pathway analysis on this network to obtain the main mechanisms of TWH-treated RA disease. The network details are provided in Figure S3 and Tables S18 and S19.

**Figure 5:**
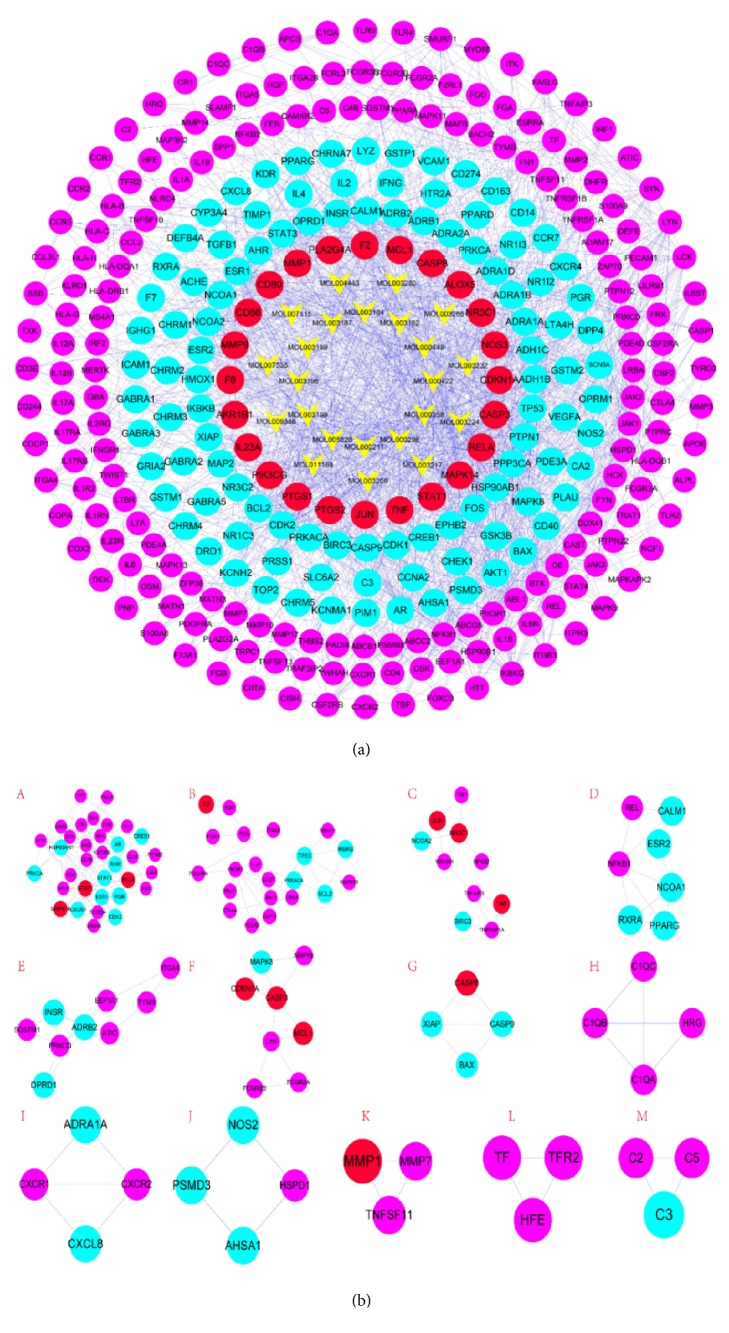
(a) TWH-RA disease network and deletions do not interact with targets and compounds (star is on behalf of TWH compounds, red circle represents compounds directly affect the RA targets, blue circle represents compound indirect role in RA targets of targets, purple is on behalf of TWH indirectly targets). (b) Cluster of compound RA disease network (A, B, C, and so on stand for clusters 1, 2, 3, and so on).

We also only deleted the drug compounds that did not directly relate the target to obtain the C-D target intersection network for later analysis. The network details are provided in Figure 5(a) and Tables S20 and S21.

#### 3.3.2. Clusters of TWH-Rheumatoid Arthritis Network

The cluster analysis method is the same as the PPI network clustering method, and the following thirteen different categories are obtained (Figure 5(b) and Table 2) and enrichment analysis is carried out for targets with cluster score values >2.

The other enrichment analysis is the same as the above analysis method. We have obtained biological processes, molecular functions, and cell components for different clusters of targets. The details of the enrichment analysis process are provided in Table S22.

Cluster analysis of TWH's role in the major genes of RA disease can be understood that RA treatment of RA can inhibit angiogenesis, regulate innate immune response, and adapt to immune response and inhibit migration and invasion of fibroblast-like synoviocytes and other ways. Now research shows that Tripterygium can also treat RA through the above-mentioned effects.

The effect of TWH on RA was not inferior to methotrexate, and its adverse reaction was slightly lower than methotrexate [26]. The latest research shows that TWH can inhibit RA by a variety of ways to inhibit angiogenesis. First, TWH can downregulate the expression of mRNA in VEGF of cells to inhibit the production of VEGF, which leads to a decrease in the content of VEGF in chondrocytes [27]. Moreover, TWH can also inhibit macrophages secreting VEGF into blood vessels to reduce local inflammatory tissue angiogenesis. In addition, TWH can inhibit the formation of AP-1 binding protein complex and inhibit the expression of VEGF. Finally, TWH can also reduce VEGF levels by inhibiting angiogenic activation factors such as TNF-*α* and IL-6 [28].

The migration and invasion of fibroblast-like synoviocytes (FLS) to cartilage and bone cells are critical processes for the destruction of RA cartilage. TWH can downregulate the expression levels of IL-32, MMP-1, and MMP-9 in IL-1*β*-stimulated RSC-364 cells (rat synoviocyte line) and inhibit cartilage breakage. In the collagen-induced severe combined immunodeficient mouse model of RA, triptolide can reduce the activation of TNF-*α* and MMP-9 to inhibit the expression of JNK signaling pathway and specifically inhibit the migration of RA FLS cells and improve joint inflammation and cartilage breakout in RA mice [29].

In the TWH test on proliferation of splenic lymphocytes, the triptolide group showed dose-dependent inhibition of Con A-induced proliferation of T lymphocytes and inhibited LPS-induced proliferation of B lymphocytes. This suggests that the effect of TWH on RA treatment is related to inhibition of T lymphocyte proliferation activity [30]. The ratio of CD4^+^/CD8^+^ was significantly higher in RA patients compared with normal controls, and the levels of B-cells and plasma lipid peroxides (LPO) were also significantly higher than those in normal subjects. After one month of treatment with TWH, the patient's clinical symptoms were significantly relieved. The ratio of CD4^+^/CD8^+^, B cell, and plasma LPO levels were significantly lower than before treatment. CD4^+^ is a helper/inducing cell (Th/Ti) that has the function of helping T lymphocytes to transform into effector cells, B cells to generate antibodies and macrophage activation, and plays a role in assisting and inducing cellular and humoral immunity; CD8+ cells are T-inhibitory/Killer cells (Ts/CTL) having the effect of inhibiting T cell activation, inhibiting B cell production of antibodies and cytotoxicity, and inhibiting cellular and humoral immunity [31]. This shows that TWH has a dual role in inhibiting cellular and humoral immunity.

### 3.4. TWH-Rheumatoid Arthritis –Mechanism Network

**Figure 6:**
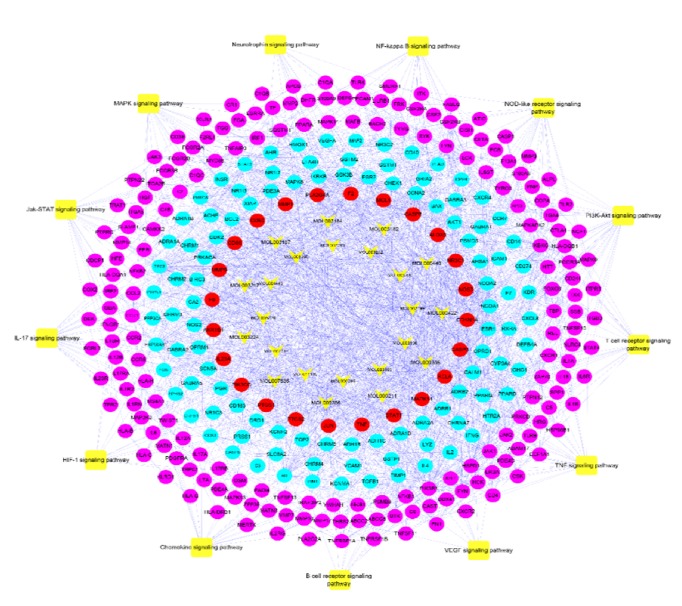
Pathway for TWH-Rheumatic Arthritis network only includes intersection targets (the yellow square represents the target action pathway, red circle represents compounds directly affect the RA targets, blue circle represents compound indirect role in RA targets of targets, purple is on behalf of TWH indirectly targets).

Drug compounds associated with RA genes were placed in the DAVID for pathway analysis and Cytoscape was used to establish compound target–pathway network (Figure S4 and Figure 6). There are 115 nodes and 248 edges on this network. This network includes 13 pathways and 102 RA genes that interact with TWH compounds. PI3K-Akt signaling pathway contains most genes (31 genes), IL-17 signaling pathway has 27 genes, TNF signaling pathway has 24 genes, NOD-like receptor signaling pathway and Jak-STAT signaling pathway have 23 genes, and MAPK signaling pathway has 21 genes. Chemokine signaling pathway has 20 genes, 19 genes of T cell receptor signaling pathway and 19 genes of NF-kappaB signaling pathway and so on. These pathways may play a crucial role in the treatment of RA by TWH drugs. The details are described in Tables S23 and S24.

The PI3K-Akt signaling pathway in RA is involved in the promotion of aggressive immune-cell and synovial cell proliferation and survival, neovascularization, apoptosis, and altered innate immunity. It can affect the increase of chemotaxis of immune cells such as neutrophils, macrophages and eosinophils, degranulation of mast cells, and activation, maturation, and survival of activated T cells and B cells. At the same time, this pathway can also regulate some cytokines such as VEGF and FGF factors to induce apoptosis and survival of synovial cells, chondrocytes, and immune cells [32, 33].

The NOD2 signaling pathway mainly regulates the innate immune system and plays an important role in eliminating infection and avoiding excessive tissue damage. NOD2 can attenuate TNF-*α*/IFN-*γ*-induced apoptosis and regulate a series of anti-inflammatory genes such as IL-10. NOD2 can also be regulated to adapt to the immune system [34]. It is produced by the key drivers of T-helper (Th) type 2 immunity, such as IL-4 and IL-5. NOD2 was highly expressed in RA tissues, and downregulation of NOD2 significantly reduced the levels of proinflammatory cytokines, NF-*κ*B, TRAF6, and IKK [35].

The IL-17 signaling pathway and TNF signaling pathway are considered classical immune signaling pathways, which can activate the intracellular molecular signal of RA pathogenesis. The route of delivery leads to the activation of endoplasmic reticulum cells and recruitment of congenital and adaptive immune cells to synovial cells, resulting in inflammation of synovial cells, increased angiogenesis, and decreased lymphangiogenesis. For these cytokine inhibitors have been used clinically or in clinical trials [36–38].

The MAPK family includes members such as tyrosine phosphoprotein kinase (p38), c-Jun N-terminal kinase (JNK), and extracellular-signal regulated protein kinase (ERK 1/2). In synovial tissue of RA, p38 is highly expressed and activated. The commonly used p38 MAPK inhibitor SB203580 reduces the production of proinflammatory cytokines in monocytes/macrophages, neutrophils, and T lymphocytes. JNK plays a pivotal role in the induction of cytokines and matrix metalloproteinase (MMP) gene expression in synovial cells. The synovial cells mainly include two subtypes of JNK1 and JNK2. Insufficiency of NK2 is only evident in preclinical models of arthritis, but JNK1 deficiency can reduce synovial inflammation and joint destruction. JNK1 also facilitates osteoclast differentiation because the progenitor cells of osteoclasts deficient in JNK1 are immature, unable to undergo bone resorption and differentiate into osteoclasts. The role of ERK in RANKL-mediated osteoclast differentiation is also crucial. The destruction of the osteoblast lineage ERK1 and ERK2 results. Osteoclasts are produced by the nuclear stimulating factor receptor (RANKL), which in turn causes osteoclasts to decrease [39, 40].

The network can see the relationship between the compound and the target, and the target interacts with the gene. However, due to the complex intracellular relationships between molecules, this network analysis is too complex. For example, MOL000528 compounds directly act on 3 RA-related genes and 31 indirectly on RA genes. These 31 genes act on 82 RA-related genes, and all targets also act on 8 major cell signaling pathways.

**Figure 7:**
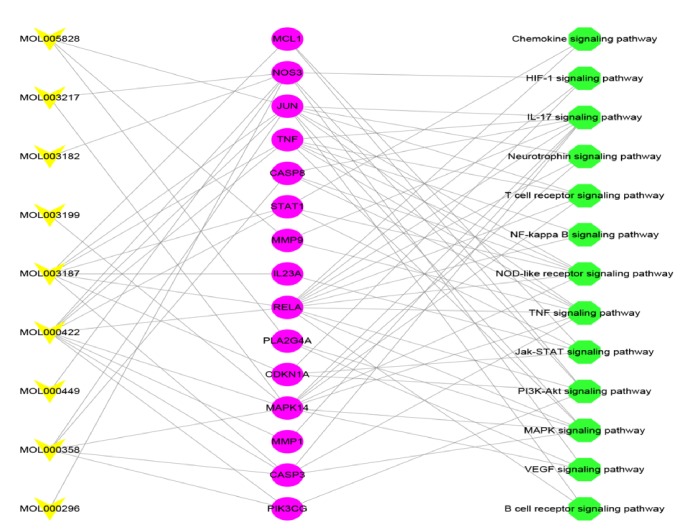
The pathway of direct action target of TWH (the yellow is the compound, the magenta is the target, the green is the signaling pathway, MOL0005828 represents nobiletin compound, MOL003217 represents Isoxanthohumol compound, MOL003182 represents (+)-Medioresinol di-O-beta-D-glucopyranoside_qt compound, MOL003199 represents 5,8-Dihydroxy-7-(4-hydroxy-5-methyl-coumarin-3)-coumarin compound, MOL003187 represents triptolide compound, MOL000422 represents kaempferol compound, MOL000449 represents Stigmasterol compound, MOL000358 represents beta-sitosterol compound, MOL000296 represents hederagenin compound.)

In order to better and concisely represent the mechanism of action of TWH compounds in the treatment of RA, we deleted indirect target and indirect acting genes, resulting in target interactions and pathway maps between TWH compounds (Figure 7). The network node is significantly less than the previous network, making the amount of network information more concise and easy to analyze. This network contains only 9 compounds, 15 targets, and 13 pathways. The details are described in Tables S27 and S28.

JUN is an important node of the JNK signaling pathway, which is widely involved in the regulation of cell proliferation, differentiation, apoptosis, metabolism, movement, and DNA damage repair [41, 42]. In our study, the JUN gene can be directly affected by Mol005828, Mol003187, Mol000422, and Mol000358 compounds in Figure 7. The gene is enriched in seven pathways. The enrichment pathways are, respectively, the B cell receptor signaling pathway, IL-17 signaling pathway, and MAPK signaling pathway, Neurotrophin signaling pathway, T cell receptor signaling pathway, NOD-like receptor signaling pathway, TNF signaling pathway. Tetrandrine inhibits the migration and invasion of RA fibroblast-like synoviocytes by affecting the JNK signaling pathway [43]. JNK-dependent regulation of mRNA stability serves as an important PKB-independent mechanism for cytokine regulation of FoxO1 and suggests that reduced FoxO1 expression is required to promote FLS survival in RA [44].

In RA, nitric oxide (NO) is involved in inflammation, angiogenesis, and tissue destruction. Nitric oxide synthase regulates nitric oxide synthesis in the body [45]. Our study showed that NOS3 can be directly affected by MOL000296, MOL000422, MOL000449, MOL003182, and MOL003217 compounds and the gene was enriched in the three pathways of HIF-1 signaling pathway, PI3K-Akt signaling pathway, and VEGF signaling pathway. In patients with rheumatic diseases, high levels of NO, NO is associated with tissue hypoxia. Nitric oxide (NO) regulates T cell function under physiological conditions, but overproduction of NO may result in T lymphocyte dysfunction [46]. Increased NO in a mouse model of bacterial infection can upregulate proinflammatory cytokines like collagen, trigger NO production, drive hypoxia, and induce synovial angiogenesis and hyperplasia to synovial injury [47].

Caspase-3 (CASP-3) is known to cause apoptosis in cells. The expression of caspase-3 mRNA in PBLs was significantly higher in MT-treated RA patients than in patients without MT [48]. Our study showed that CASP3 can be directly affected by MOL000422 and MOL003187 compounds and the gene was enriched in the three pathways of IL-17 signaling pathway, MAPK signaling pathway, and TNF signaling pathway. The caspase-8(CASP-8) protein involves functions such as apoptosis and influencing innate immunity [49]. CASP8 can be directly affected by MOL000358 compound and the gene was enriched in the three pathways of IL-17 signaling pathway, NOD-like receptor signaling pathway, and TNF signaling pathway.

Mcl-1 is a Bcl-2 family of antiapoptotic molecules that are essential for the survival of T and B lymphocytes and macrophages and also is essential for the survival of synovial fibroblasts in RA [50]. Our study showed that MCL1 can be directly affected by MOL003187 compound and the gene was enriched in the two pathways of Jak-STAT signaling pathway and PI3K-Akt signaling pathway. Mcl-1 is essential for the survival of intra-articular macrophages in RA patients. Compared with normal in vitro differentiated macrophages, the expression of Mcl-1 in CD14^+^ macrophages in the synovial fluid of RA patients increases. Inhibition of the PI3-kinase/Akt-1 or STAT-3 pathway significantly reduced the percentage of CD14^+^ cells within the synovial fluid and led to a decrease in Mcl-1 and induction of synovial macrophage apoptosis. Transfection of RA synovial macrophages with Mcl-1 siRNA resulted in apoptotic cell death [51].

The phospholipase A2 group IVA gene (PLA2G4A) encodes a member of the cytoplasmic phospholipase A2 group IV family. The enzyme catalyzes the hydrolysis of membrane phospholipids to release arachidonic acid, which is then metabolized to eicosanoids. Eicosanoids including prostaglandins and leukotrienes are lipid-based cytokines that regulate hemodynamics, inflammatory responses, and other intracellular pathways. The hydrolysis reaction also produces lysophospholipids that are converted into platelet activating factors. The enzyme is activated by increasing intracellular Ca^2+^ levels and phosphorylation, resulting in its translocation from the cytoplasm and nucleus to the perinuclear membrane vesicles. PLA2G4A is regulated by MOL005828 compound, which is enriched in MAPK signaling pathway and VEGF signaling pathway. Oral pyrrole administration achieves antiarthritis activity by inhibiting cPLA2*α* activity, resulting in decreased eicosanoid levels and inhibition of MMP and COX-2 mRNA expression. These results support the potential therapeutic role of alpha in the treatment of RA by the alpha 2 inhibitor of cPLA [52].

The MMP1, MMP9, MAPK14, TNF, and PIK3CG proteins in the previous discussion can affect the inflammatory response system, affecting processes such as apoptosis, cell cycle, and immune cell migration. Our study showed that MMP1 can be directly affected by MOL000422 compound and the gene was enriched in the pathway of IL-17 signaling pathway. The MMP9 was regulated MOL005828 and the gene was enriched in the two pathways of IL-17 signaling pathway and TNF signaling pathway. MAPK14 is regulated by four compounds (MOL000358, MOL000422, MOL003199, and MOL003217) and accumulated to seven pathways in the enrichment analysis of IL-17 signaling pathway, MAPK signaling pathway, and Neurotrophin signaling pathway, NOD-like receptor signaling pathway, T cell receptor signaling pathway, TNF signaling pathway, and EGF signaling pathway. TNF by MOL000422 and MOL003187 is regulated by two compounds and accumulated to six pathways in the enrichment analysis of IL-17 signaling pathway, MAPK signaling pathway, NF-kappa B signaling pathway, NOD-like receptor signaling pathway, T cell receptor signaling pathway, and TNF signaling pathway. PIK3CG is regulated by MOL000358 and MOL000422 compounds and this gene is enriched in PI3K-Akt signaling pathway.

We found that different compounds in TWH play different roles in the treatment of RA. MOL000422 is the compound that TWH has the most target for RA. It acts on 13 pathways through 8 targets (NOS3, JUN, TNF, STAT1, REAL, MAPK14, CASP3, and PZK3CG). MOL003187 compounds act on 12 RA-related pathways via the 8 targets (MCL1, JUN, TNF, STAT1, REAL, IL23A, CASP3, and CDKM1A). MOL003217 compound directly act on the NOS3 and MMK14 target, and these two targets act on 9 pathways. MOL005828 compound directly acts on JUN, MMP9, and PLA2G4A3 targets, and these three targets act on 8 pathways. MOL003199 compound acts on 7 RA-related pathways via the MAPK14 targets. MOL000358 compound acts on 9 RA-related pathways via the 5 targets (JUN, CASP8, MAPK14, CASP3, and PZK3CG). Some compounds act together on the same target and act on the same pathway, such as MOL000296, MOL004449, and MOL003182 and simultaneously act on F1F-1, PI3K-AKT, and VEGF signaling pathways through NOS3 target.

## 4. Conclusions

Our study found that some compounds in TWH can directly act on relevant targets to exert anti-inflammatory effects, such as luteolin, quercetin, and naringenin, etc., and some compounds indirectly exert anti-inflammatory effects through their targets, such as Triptonolide, Tryptophenolide, and Zhebeiresinol, etc. In this study, by collecting TWH compounds and their properties, screening active compounds, collecting targets, and disease-related targets, and constructing a variety of pharmacological networks we predict, explore, and clarify the molecular cooperation mechanism of TWH in the treatment of RA. We have also successfully identified targets related to RA, clusters of targets, biological processes of targets, molecular functions, and cell components and related pathways. The construction of the pathway of RA disease protein-protein interactions reveals the complex underlying molecular interaction mechanisms of RA. Finally, the C-D-Target network and the pathway of C-D-Target network were constructed to analyze, confirm, and reveal the potential pharmacological and molecular mechanisms of TWH in the treatment of RA.

## Figures and Tables

**Table 1:** Cluster of rheumatoid arthritis disease PPI network.

Cluster	Score	Nodes	Edges	GENES
1	3.375	31	54	LTA, FGB, FGG, SYK, TLR4, TBP, TLR9, ITGB3, MYD88, ADAM17, NFKB2, PDGFRA, PLA2G4A, RELA, TNFRSF1A, CTLA4, REL, C1QA, HRG, C1QB, C1QC, JAK2, NCF1, PRKCD, F13A1, IRF1, STAT1, F2, IRF2, TNFRSF1B, LCK

2	2.4	4	6	CASP8, IKBKG, TNF, TNFAIP3

3	2.333	5	7	HLA-B, HLA-C, LILRB1, HLA-H, HLA-G

4	2.25	7	9	PTPRC, FCGR3A, CD4, CD3E, PTPN22, PIK3R1, CSK

5	2.2	9	11	ABL1, TNFSF11, MMP7, JAK1, CCL2, SMURF1, MMP1, BTK, CCR1

6	1.5	3	3	HFE, TFR2, TF

7	1.5	3	3	IL17RA, TRAF3IP2, IL17A

**Table 2:** Clusters of TWH-Rheumatoid arthritis network.

Cluster	Score	Nodes	Edges	GENES
1	6.1	35	110	CCR5, JAK2, IKBKG, HTT, HSP90AB1, HCK, FYN, FOS, CSF2RB, CREB1, IRF2, BTK, AR, S100A8, RELA, ITK, ESR1, AHR, FASLG, STAT3, MAPK14, CDK2, CSK, FCGR3A, CD4, PTPN22, JAK3, STAT1, SYK, PRKCA, CD3E, IL6ST, CXCR4, PGR, FN1

2	3.1	21	34	FGG, ABL1, PIK3R1, ZAP70, TP53, PRKACA, CTLA4, PDE4D, MAPK11, LRBA, ITGB3, TWIST1, BCL2, IKBKB, TRAT1, F2, LCK, PDGFRA, CAST, FGB, F13A1

3	2.9	10	16	NCOA2, TNFRSF1A, TNF, TNFAIP3, BIRC3, NR3C1, YWHAH, NFKB2, JUN, TBP

4	2.7	7	11	CALM1, NCOA1, RXRA, NFKB1, ESR2, REL, PPARG

5	2.6	9	13	TYMS, ADRB2, ATIC, OPRD1, ITGA4, SQSTM1, PRKCD, INSR, EEF1A1

6	2.4	8	11	LYN, CDKN1A, FCGR2B, FCGR2A, CASP3, MAPK9, MAPK8, MCL1

7	2.4	4	6	CASP8, BAX, CASP9, XIAP

8	2.0	4	5	HRG, C1QC, C1QB, C1QA

9	2.0	4	5	CXCR1, ADRA1A, CXCL8, CXCR2

10	1.6	4	4	HSPD1, NOS2, PSMD3, AHSA1

11	1.5	3	3	MMP7, MMP1, TNFSF11

12	1.5	3	3	TFR2, TF, HFE

13	1.5	3	3	C5, C3, C2

## Data Availability

The supplementary data associate with “A network pharmacology study on the active ingredients and potential targets of* Tripterygium wilfordii *Hook for treatment of rheumatoid arthritis” used to support the findings of this study are included within the supplementary information files. The valid supplementary material files were listed in appendix of email.

## Supplementary Materials

Supplementary Materials Composite compounds of TWH (Table S1). Compounds with an oral bioavailability value greater than 30% (Table S2). Compounds with a Caco-2 value > -0.3 (Table S3). Compounds with a drug-likeness value >0.18 (Table S4). Compound with a half-life of 3 hours (Table S5). The potential bioactive compounds of TWH using an in silico integrative model ADME (Table S6). The therapeutic target data for RA treatment from four resources, including TDD, OMIM, PHARMGKB, and GADA (Table S7). Detailed information on six existing protein-protein interaction databases (Table S8). Prediction of putative targets for compositive compounds Tripterygii Radix (Table S9). Four topological feature values of targets in target-compositive compounds network (Table S10). Rheumatoid Arthritis disease PPI Network (Table S11). Four topological feature values of rheumatoid arthritis disease PPI network (Table S12). The enrichment analysis of the target cluster analysis of rheumatoid arthritis (Table S13). Pathway of Rheumatic Arthritis target (Table S14). Four topological feature values of Pathway with Rheumatic Arthritis target (Table S15). TWH-Rheumatic Arthritis network (Table S16). Four topological feature values of TWH-Rheumatic Arthritis network (Table S17). TWH-Rheumatic Arthritis network and only including intersection targets (Table S18). Four topological feature values of TWH-Rheumatic Arthritis network and only including intersection targets (Table S19). TWH-Rheumatic Arthritis network includes TWH active ingredients and intersection targets (Table S20). Four topological feature values of TWH-Rheumatic Arthritis network include TWH active ingredients and intersection targets (Table S21). Cluster of pathway for TWH-Rheumatic Arthritis network and only including intersection targets (Table S22). Pathway for TWH-Rheumatic Arthritis network and only including intersection targets (Table S23). Four topological feature values of pathway for TWH-Rheumatic Arthritis network and only including intersection targets (Table S24). Pathway for TWH- Rheumatic Arthritis network and only including intersection targets after deleting indirect target and indirect acting genes (Table S25). Four topological feature values of pathway for TWH-Rheumatic Arthritis network and only including intersection targets after deleting indirect target and indirect acting genes (Table S26). Rheumatoid arthritis disease target PPI Network (Figure S1). Rheumatoid arthritis disease target PPI Network (Figure S2). TWH-RA disease network and only including targets for disease (Figure S3). Pathway of TWH-RA (Figure S4).
