# Supplementary material for: A Network Pharmacology Study on the Active Ingredients and Potential Targets of Tripterygium wilfordii Hook for Treatment of Rheumatoid Arthritis
Source: Evid Based Complement Alternat Med. 2019 Apr 15;2019:5276865. doi: 10.1155/2019/5276865 (PMC6500618; doi:10.1155/2019/5276865)
Supplement: Supplementary Materials — Composite compounds of TWH (Table S1). Compounds with an oral bioavailability value greater than 30% (Table S2). Compounds with a Caco-2 value > -0.3 (Table S3). Compounds with a drug-likeness value >0.18 (Table S4). Compound with a half-life of 3 hours (Table S5). The potential bioactive compounds of TWH using an in silico integrative model ADME (Table S6). The therapeutic target data for RA treatment from four resources, including TDD, OMIM, PHARMGKB, and GADA (Table S7). Detailed information on six existing protein-protein interaction databases (Table S8). Prediction of putative targets for compositive compounds Tripterygii Radix (Table S9). Four topological feature values of targets in target-compositive compounds network (Table S10). Rheumatoid Arthritis disease PPI Network (Table S11). Four topological feature values of rheumatoid arthritis disease PPI network (Table S12). The enrichment analysis of the target cluster analysis of rheumatoid arthritis (Table S13). Pathway of Rheumatic Arthritis target (Table S14). Four topological feature values of Pathway with Rheumatic Arthritis target (Table S15). TWH-Rheumatic Arthritis network (Table S16). Four topological feature values of TWH-Rheumatic Arthritis network (Table S17). TWH-Rheumatic Arthritis network and only including intersection targets (Table S18). Four topological feature values of TWH-Rheumatic Arthritis network and only including intersection targets (Table S19). TWH-Rheumatic Arthritis network includes TWH active ingredients and intersection targets (Table S20). Four topological feature values of TWH-Rheumatic Arthritis network include TWH active ingredients and intersection targets (Table S21). Cluster of pathway for TWH-Rheumatic Arthritis network and only including intersection targets (Table S22). Pathway for TWH-Rheumatic Arthritis network and only including intersection targets (Table S23). Four topological feature values of pathway for TWH-Rheumatic Arthritis network and only including inters [file 5276865.f1.zip › Supplementary Materials/Supplementary Figures.docx]

**A** **netwo****rk pharmacology study on the active ingredients and potential targets of Tripterygium wilfordii Hook for treatment of rheumatoid arthritis**

Wei Hu ^1,2^, Wanjin Fu ^2^, Xin Wei ^2^, Yang Yang ^2^, Chao Lu ^2,^*, Zeyuan Liu ^1,^*

1. Department of Clinical Pharmacology, Affiliated Hospital of Academy of Military Medical Sciences, Beijing, China
2. Department of Pharmacology, The Second Hospital of Anhui Medical university, Hefei, Anhui, China

*Correspondence to: Professor Chao Lu or Zeyuan Liu, E-mail: 765385306@qq.com; E-mail: [13701359937@163.com](mailto:13701359937@163.com).


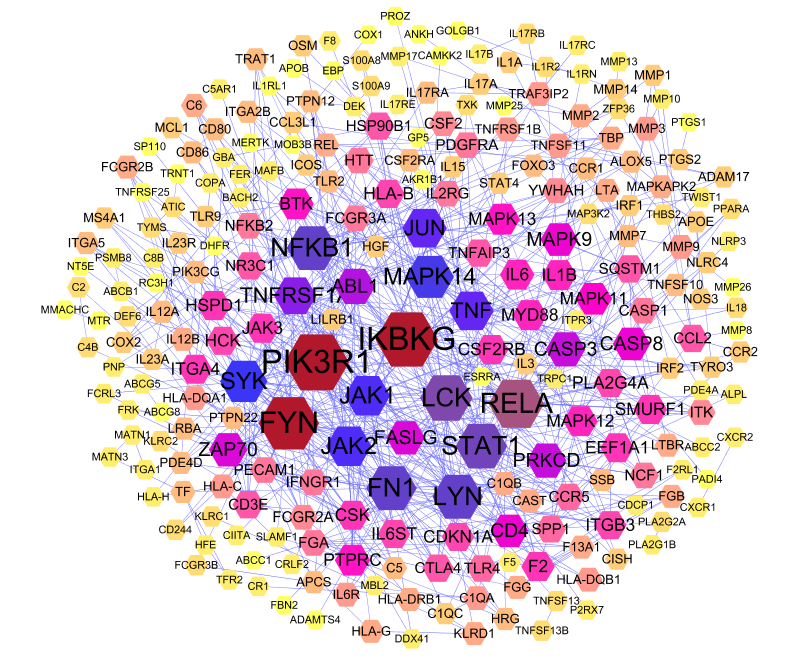


Figure S1. Rheumatoid arthritis disease target PPI Network.


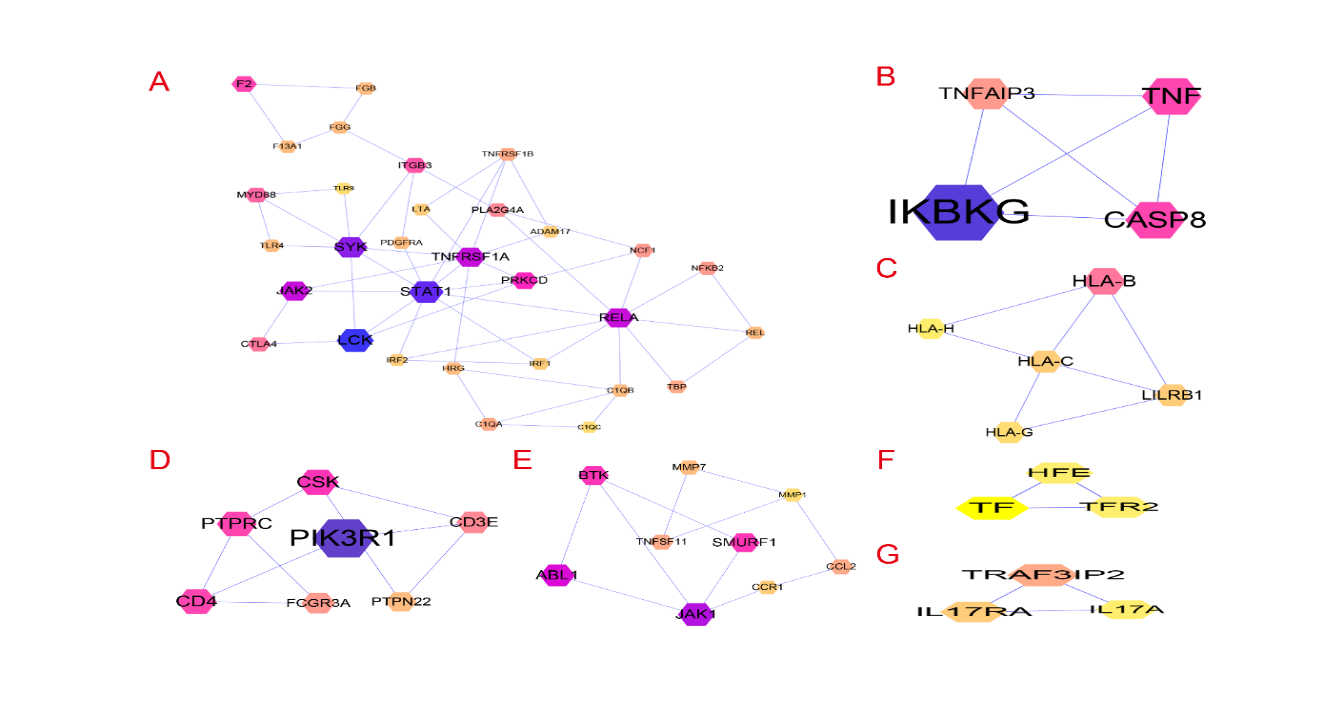


Figure S2. Rheumatoid arthritis disease target PPI Network (A, B, C,D,E,F,G stand for cluster 1, 2, 3,4,5,6,7).


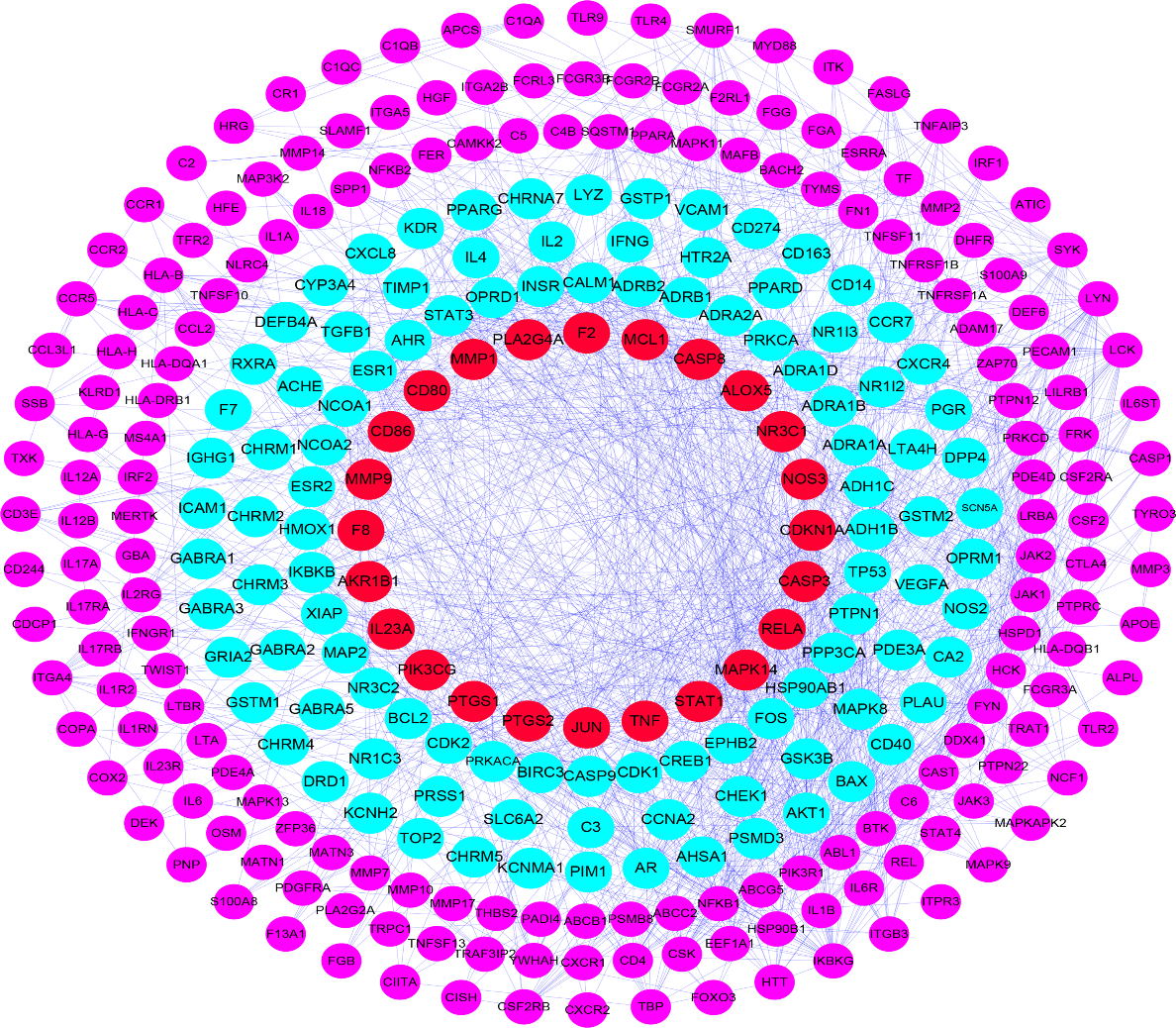


Figure S3.TWH -RA disease network and only includes targets for disease. (red circle represents compounds directly effect the RA targets, blue circle represents compound indirect role in RA targets of targets, purple on behalf of TWH indirectly targets).


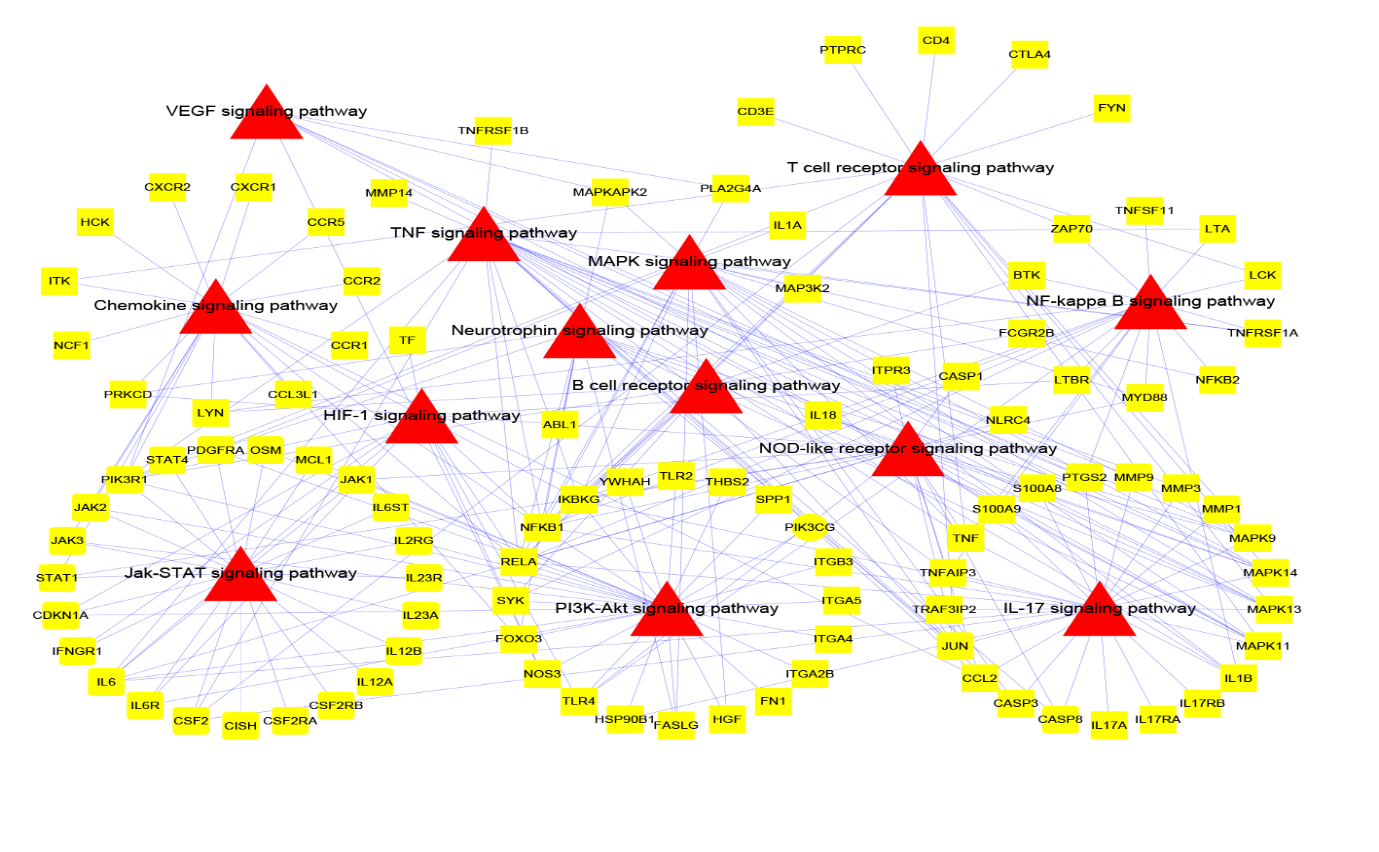


Figure S4. Pathway of TWH-RA (The triangle represents the path and the square represents the target.)
